# The Case for Abandoning Therapeutic Chelation of Copper Ions in Alzheimer's Disease

**DOI:** 10.3389/fnins.2017.00317

**Published:** 2017-06-02

**Authors:** Simon C. Drew

**Affiliations:** Department of Medicine, Royal Melbourne Hospital, University of MelbourneMelbourne, VIC, Australia

**Keywords:** Alzheimer's disease, β-amyloid, copper, bioinorganic chemistry, N-truncation, chelator, metals hypothesis, metal homeostasis

## Abstract

The “therapeutic chelation” approach to treating Alzheimer's disease (AD) evolved from the metals hypothesis, with the premise that small molecules can be designed to prevent transition metal-induced amyloid deposition and oxidative stress within the AD brain. Over more than 20 years, countless *in vitro* studies have been devoted to characterizing metal binding, its effect on Aβ aggregation, ROS production, and *in vitro* toxicity. Despite a lack of evidence for any clinical benefit, the conjecture that therapeutic chelation is an effective approach for treating AD remains widespread. Here, the author plays the devil's advocate, questioning the experimental evidence, the dogma, and the value of therapeutic chelation, with a major focus on copper ions.

## Introduction

The “amyloid cascade hypothesis” of Alzheimer's disease (AD) proposes disease is caused by accumulation of the β-amyloid (Aβ) peptide (typically up to 42 residues in length) that is proteolytically derived from the amyloid precursor protein (APP; Hardy and Higgins, 1992; Masters and Selkoe, 2012). Consistent with the structure of the plaque core and congophilic angiopathy observed in post-mortem AD brain, synthetic Aβ_1−x_ (x = 28–43) peptides have a propensity to adopt β-sheet structure in aqueous solution in the pH range 4–7 (Barrow and Zagorski, 1991). Aβ_1−x_ elicits both neurotrophic and neurotoxic actions (Whitson et al., 1989; Yankner et al., 1990; Collins et al., 2015). Despite some potential experimental artifacts (Watt et al., 2013; Welzel et al., 2014) and some good arguments that the link between Aβ and AD is indirect (Herrup, 2015), soluble Aβ oligomers are widely viewed as toxic intermediates responsible for AD pathology (McLean et al., 1999; Selkoe, 2008) and sporadic AD has been associated with an inefficient clearance of Aβ from the central nervous system (Mawuenyega et al., 2010).

The “metals hypothesis of AD” argues that accumulation of Aβ is insufficient to explain the onset of AD and that dysregulation of the brain's intrinsic supply of metal ions, notably copper, zinc, and iron, creates a “rogue” form of Aβ that promotes aggregation and, in the case of copper and iron, generates reactive oxygen species (ROS) that drive disease (Bush et al., 1994a; Bush, 2000, 2003, 2008; Bush and Tanzi, 2008; Duce et al., 2011).

From the perspective of the bioinorganic chemist, the past decade has led to a reasonable consensus regarding the coordination chemistry, thermodynamic stability and *in vitro* mechanism of ROS production by copper and Aβ (Drew and Barnham, 2011; Faller et al., 2014; Reybier et al., 2016). Despite this detailed knowledge, no therapeutic has been designed that specifically takes advantage of this structural information, although a large number of chelators and oligopeptides have been proposed (reviewed in Telpoukhovskaia and Orvig, 2013). Perhaps, then, it is not surprising that clinical trials of metal chelators have suffered the same set-backs as anti-amyloid therapies. While this may reflect an inappropriate choice of chelator, it also raises the question as to the validity of the underlying hypothesis, especially given the number of controversial findings over the past 20 years. Some examples include the assertions that: Aβ possesses a superoxide dismutase-like di-copper binding site (Curtain et al., 2001; Tickler et al., 2005; Smith et al., 2006); Aβ generates ROS in the absence of metal ions (Hensley et al., 1994; Turnbull et al., 2001); Met35 reduces Cu^2+^(Aβ) to an air-stable Cu^+^(Aβ) complex (Barnham et al., 2003a; Ciccotosto et al., 2004); Cu^2+^ cannot displace Zn^2+^ from Aβ_1−40_ (Bush et al., 1994b); the affinity of Aβ_1−42_ for Cu^2+^ is 10 attomolar, seven orders of magnitude higher than that of Aβ_1−40_ (Atwood et al., 2000); rat and mouse Aβ bind Cu^2+^ much less avidly than human Aβ (Bush et al., 1994a; Atwood et al., 1998; Eury et al., 2011); Aβ does not aggregate in the absence of metal ions (Atwood et al., 2000; Bush and Tanzi, 2002); Aβ plaques are “galvanized” (Bush and Tanzi, 2002);^1^ and that a 22-residue domain within APP has biological ferroxidase activity modulated by Zn^2+^ binding (Duce et al., 2010; Ebrahimi et al., 2012, 2013).

The above examples aside, there has also been a change in focus in recent years from metal ion interaction with Aβ as a driver of aggregation and toxicity to a more general picture of global metal ion dysregulation, in which direct metal-Aβ interactions play a secondary or even inconsequential role (White et al., 2006; James et al., 2012; Singh et al., 2013). In other instances, there has been a synthesis of the two schools of thought, whereby an accumulation of metal ions in amyloid plaques is proposed to be responsible for the loss of normal metal ion balance (Hung et al., 2011; Ceccom et al., 2012; Roberts et al., 2012; Ayton et al., 2013). With respect to copper ions, some propose that AD is a disease of dietary copper deficiency (Klevay, 2008), while others propose it is caused by excess inorganic copper in the diet that can be treated using zinc therapy (Hoogenraad, 2011; Brewer, 2014) or a low-copper diet (Squitti et al., 2014b). Soluble, monomeric Aβ_1−x_ has even been proposed to possess a normal function in metal export, whereby metal-enrichment within plaques is associated with a loss of function (Kepp, 2016). Other potential physiological functions of Aβ_1−40/42_ have been proposed, as an antimicrobial peptide (Soscia et al., 2010; Kumar et al., 2016) and as a cerebrovascular sealant (Atwood et al., 2003), although any role for copper ions in these contexts remains to be established.

## Is biological Aβ metal binding feasible?

Four common arguments made in favor of the biological/disease relevance of metal-Aβ interactions are (i) the release of “high” concentrations of copper and zinc ions in the synaptic cleft upon depolarization, 15–250 μM in the case copper (Kardos et al., 1989; Hartter and Barnea, 1998); (ii) Zn^2+^, Cu^2+^, and Fe^3+^-induced aggregation of synthetic Aβ (Atwood et al., 1998; Cherny et al., 1999), (iii) elevated concentrations of Aβ within the AD cortex as compared with unaffected individuals (Lue et al., 1999); and (iv) a “high” affinity of human Aβ for Cu^2+^. The apparent dissociation constant of Aβ_1−x_ (x ≥ 16) at pH 7 is ~0.1 nM (Alies et al., 2013; Young et al., 2014) and thus better described as “moderate,” since other metal-binding species with comparable (or higher) capacity to bind Cu^2+^ are also present in the central nervous system (CNS). For example, glutamate also reaches transiently high local concentration during synaptic signaling (Danbolt, 2001), making it competitive at relevant physiological concentrations (Frączyk et al., 2016), while other neurotransmitters such as histamine (HA) have even higher Cu^2+^ affinity than glutamate (Dawson et al., 1990). Aside from one study concluding Aβ_1−42_ oligomers have an enhanced affinity (*K*_d_ < 3 pM in HEPES pH 7.4; Jiang et al., 2013) that enables them to compete with human serum albumin (HSA), the latter has also been proposed as a major competitor with Aβ for copper ions within the CNS (Rózga and Bal, 2010), effectively competing for 99.9% of Cu^2+^ (Perrone et al., 2010) and binding Cu^+^ stronger than Aβ (Lu et al., 2015). Metallothioneins (MTs) are likely competitors for extracellular Cu^2+^ in the CNS (Meloni et al., 2008; Chung et al., 2010). Moreover, the endoproteolytic cleavage product Aβ_4−x_, which is also present in healthy cortex (see below), has very high affinity (Mital et al., 2015) and can even retain Cu^2+^ in competition with MTs (Wezynfeld et al., 2016).

The pecking order, even among this limited selection of Cu^2+^ binding species, is therefore likely Aβ_4−x_ ≥ MTs > HSA > Glu, HA > Aβ_1−x_. To explain why Aβ_1−x_ interacts with synaptic Cu^2+^ only in disease, one must therefore argue that there exists an underlying imbalance that creates abnormalities in the regulation of metal-binding amino acids, peptides and proteins. For example, the concept of the “labile copper pool” has been introduced (James et al., 2012). This places metal-Aβ_1−x_ interactions downstream of an underlying pathology, making this modest-affinity, non-specific binding non-central to disease pathogenesis. The non-specificity argues that a range of other proteins and peptides could also adopt unwanted, modest-affinity (and potentially redox-active) Cu^2+^ coordination, making the strong focus on Aβ_1−x_ unwarranted.

*In vitro* evidence for the ability of Aβ_1−x_ to generate ROS in the presence of Cu^2+^ and biological reducing agents was quickly established (Huang et al., 1999; Opazo et al., 2002) although continues to be debated (Mayes et al., 2014; Pedersen et al., 2016; Reybier et al., 2016), while the underlying coordination chemistry has largely been unraveled (Drew and Barnham, 2011; Faller et al., 2014). A greater degree of H_2_O_2_ production was reported for Cu^2+^ and Fe^3+^ in the presence of human Aβ_1−x_ vs. rat/mouse Aβ_1−x_ (Huang et al., 1999; Barnham et al., 2003a), which was concluded to be consistent with an absence of amyloid pathology in these animals. Although the latter is a common argument made in support of the metals hypothesis (Bush et al., 1994b; Atwood et al., 1998; Huang et al., 1999; Barnham et al., 2003a), aged rats can exhibit neuritic plaques (Vaughan and Peters, 1981) and a number of AD-related functional, morphological and behavioral changes are observed in wild type rats and mice if clearance of murine Aβ is impaired by the pharmacological inhibition or genetic ablation of Aβ degrading/clearing enzymes such as neprilysin (NEP) and ATP-binding cassette C1 (Iwata et al., 2000; Madani et al., 2006; Krohn et al., 2015). Since human and murine Aβ adopt rather different Cu^2+^ coordination (Eury et al., 2011), this argues against a specific role for direct Cu-Aβ interaction and instead reinforces the importance of Aβ clearance.

While exogenous application of Aβ to cultured cells appears capable of causing oxidative damage that can be prevented by metal chelators or antioxidants (Behl et al., 1992, 1994; Manelli and Puttfarcken, 1995; Rosales-Corral et al., 2012), distinguishing direct metal-Aβ redox cycling from downstream oxidative damage and cell dysfunction is not straightforward. Similar caveats apply to reports that metal chelators inhibit β-amyloid accumulation in transgenic mice (Cherny et al., 1999), since the chelator may bind metal ions released downstream of cellular events triggered by the apo-peptide. To “potentiate” the toxicity of Aβ, it is common to co-administer Cu^2+^ with Aβ to cultured cells (Smith et al., 2006; Sarell et al., 2010), frequently at relatively high steady-state concentrations up to 40 μM (Meloni et al., 2008; Chung et al., 2010; Perrone et al., 2010). Despite the corresponding Cu^2+^ alone being generally tolerable in such cell culture systems, a “double insult” cannot be ruled out, whereby Cu^2+^ compounds upstream stress caused by Aβ in isolation. The non-specific nature of this mechanism of “potentiating” toxicity of Aβ is demonstrated by the fact that similar results can be achieved by co-administering Fe^3+^ and Aβ to cultured neurons (Schubert and Chevion, 1995; Rottkamp et al., 2001), yet Aβ has an extremely low affinity for Fe^3+^ (Valensin et al., 2011), making Fe(Aβ) redox cycling unlikely.

In order to demonstrate direct binding of Cu^2+^ to Aβ within senile plaque cores, brain amyloid extractions (Selkoe et al., 1986; DeWitt et al., 1998) have been subjected to Raman spectroscopic analysis (Dong et al., 2003). Using prior Raman investigations of metal binding to synthetic Aβ (Miura et al., 2000) as a guide, Dong et al. (2003) concluded that Cu^2+^ (and Zn^2+^) were directly bound to Aβ in plaques, based upon intensity changes assigned to metal ion coordination to His side chains. Whilst highly suggestive of direct Cu^2+^-Aβ binding, the actual composition of those senile plaque extracts is not known, nor is the true origin of metal ions they contain. Purity of amyloid cores was estimated as >90% based upon Congo red birefringence (Dong et al., 2003), with further evidence of purity including the inability to observe a Raman band due to Trp (Aβ contains no Trp). Given the possibility of up to 10% impurities and the fact that Trp is the lowest frequency amino acid observed in vertebrates, one simple explanation for the Cu^2+^-His Raman bands is that they derive from non-amyloid components. Regardless of the limitations in final purity by this method (Rostagno and Ghiso, 2009), disruption of native metal binding sites, for example by treatment of crude brain homogenates by denaturation at 97°C and 3 mM sodium azide (DeWitt et al., 1998; Dong et al., 2003), may still lead to the release of metal ions from the many hundreds of proteins that are also found within senile plaques (Drummond et al., 2017), ultimately resulting in adventitious binding to Aβ prior to pelleting and subsequent fractionation.

As mentioned above, N-truncation of Aβ can greatly enhance its Cu^2+^ binding affinity. Following the seminal sequencing of brain amyloid (Glenner and Wong, 1984), subsequent studies identified a large degree of heterogeneity at the N-terminus, with a predominance of the Aβ_4−x_ isoform in amyloid derived from subjects with AD, Down Syndrome and cerebral amyloid angiopathy (Masters et al., 1985a,b). Nevertheless, most effort has been invested in measuring and modulating levels of Aβ_1−40/42_ in the CNS as AD biomarkers (Blennow et al., 2015) and therapies (Wisniewski and Goñi, 2014; Ingelsson and Lannfelt, 2016), respectively. With hindsight, the field's dismissal of the “ragged” N-termini appears to stem from a perception that this was, at least for Aβ_4−x_, an artifact of pepsin digestion during the plaque extraction (Masters and Selkoe, 2012). A number of observations argue against this, however; Aβ_4−x_ is also detected in collagenase digests of senile plaque cores (Miller et al., 1993), in undigested amyloid extractions (Näslund et al., 1994; Sergeant et al., 2003), and in Aβ immunoprecipitated from *post mortem* brain (Lewis et al., 2006; Portelius et al., 2010). Since the initial report of >60% Aβ_4−x_ in the amyloid plaque cores within the cortex of selected AD brain samples (Masters et al., 1985b), others surveyed Aβ_4−x_ levels in larger cohorts. Näslund et al. (1994) reported lower levels of Aβ_4−x_ as compared with Aβ_1−x_ and pyrogluamate Aβ_11−x_, but Aβ_4−x_ remained consistently detected in both AD and unaffected individuals. Sergeant et al. (2003) also concluded amino-truncated isoforms represented more than 60% of all Aβ species in advanced AD and in non-demented individuals with amyloid, with comparable Aβ_1−x_ and Aβ_4−x_ levels. Lewis et al. (2006) reported that Aβ_4−42_ was the most dominant peak in mass spectrometry analyses of AD and vascular dementia samples. Mass spectrometry analyses of Portelius et al. (2010) supported this conclusion and further reported that Aβ_4−42_ and Aβ_1−42_ are dominant isoforms in the hippocampus and cortex of sporadic AD patients, as well as in the cortex of healthy controls. In fact, cortical Aβ_4−42_ levels are comparable in AD and healthy control and are much greater in the hippocampus in AD vs. control (Portelius et al., 2010).

Of the Zn-dependent endopeptidases so far identified as Aβ degrading enzymes (Saido and Leissring, 2012; Jha et al., 2015), neprilysin (NEP; Howell et al., 1995; Kanemitsu et al., 2003) and insulin degrading enzyme (IDE; Morelli et al., 2004; Grasso et al., 2011) appear capable of cleaving the Glu3-Phe4 bond to generate Aβ_4−x_ *in vitro*. An inverse correlation has been established between NEP levels and/or activity with brain Aβ levels in aging (Russo et al., 2005) and AD (Yasojima et al., 2001; Mohajeri et al., 2002; Hellström-Lindahl et al., 2008; Zhou et al., 2013). The apparent increase of Aβ_4−42_ in the hippocampus of AD subjects might be attributed to reduced endoproteolysis at C-terminal locations within Aβ, leading to aggregates of Aβ_1−x_ in which only the amino-terminal Glu3-Phe4 bond is accessible to NEP/IDE.

Synthetic Aβ_4−40_ and Aβ_4−42_ were concluded to be as toxic to cultured primary neurons as Aβ_1−42_ (Bouter et al., 2013) and mice subjected to intracerebroventricular injection of Aβ_4−42_ exhibited memory deficits that could be rescued by passive immunotherapy using antibodies targeting the N-terminus of Aβ_4−x_ (Antonios et al., 2015). Transgenic animals expressing human APP do not accumulate the N-truncated Aβ found in human brain (Kalback et al., 2002; Schieb et al., 2011). Transgenic mice specifically expressing and releasing extracellular Aβ_4−42_ displayed spatial memory deficits and marked hippocampal neuron loss. However, both of the *in vivo* models of Aβ_4−42_ toxicity bypass the physiological pathways of Aβ_4−42_ production (i.e., the Aβ_4−42_ is not endoproteolytically cleaved from APP-derived human Aβ_1−42_). Thus, the models only represent a pathological state induced by overproduction of Aβ_4−42_ and do not permit the study of any possible function Aβ_4−x_.

The reports of Aβ_4−x_ as a major isoform in the AD brain and in the cortex of unaffected individuals have some profound consequences for the metals hypothesis. The N-terminal FRH–sequence of Aβ_4−x_ endows it with the amino-terminal Cu and Ni (ATCUN) motif that creates a Cu^2+^ binding site with an affinity (*K*_d_ = 30 fM at pH 7.4 was measured for Aβ_4−16_) that is comparable to functional cuproproteins (Mital et al., 2015). This makes it more stable than Cu(Aβ_1−x_) by more than three orders of magnitude and around 100 times higher than the reported enhancement of Cu^2+^ binding affinity by Aβ_1−42_ aggregates (*K*_d_ < 3 pM; Jiang et al., 2013). Moreover, Cu^2+^ coordinated to the high affinity binding site of Aβ_4−x_ does not appear to undergo any physiologically accessible Cu^+^/Cu^2+^ redox cycle (Mital et al., 2015; Wiloch et al., 2016). These properties suggest a functional role for Aβ_4−x_ that is arguably more plausible than any other proposed for Aβ_1−x_ and copper. With this knowledge in hand, one can look to other ATCUN motifs within the Aβ sequence, since these should also possess high affinity Cu^2+^ binding sites. The Aβ_11−x_ fragment, created by β′ cleavage, contains His in the third position that, if left unmodified, could bind Cu^2+^ with comparable affinity to Aβ_4−x_ (Barritt and Viles, 2015). It will probably not do so *in vivo*, however, since its N-terminus is cyclized to the pyroglutamate form (Näslund et al., 1994) that destroys the ATCUN motif. Another ATCUN sequence is present in Aβ_12−x_, which was identified in neurofibrillary amyloid (Masters et al., 1985a), but has since received little attention.

In summary, Raman spectroscopic evidence for Cu^2+^-His coordination within senile plaque extracts is highly suggestive of direct Cu^2+^-Aβ binding, although further evidence for the purity of the isolated senile plaque cores and the origin of metal ions is warranted. Cell culture models suggest that co-administration of copper ions enhances toxicity of exogenous, synthetic Aβ_1−40/42_, yet it remains inconclusive whether this results from direct copper-Aβ_1−x_ binding and/or whether the conditions employed are representative of those at a synapse. The Aβ_4−x_ isoform produced by endoproteolytic processing of Aβ_1−x_ presents the possibility for a 3,000-fold higher affinity Cu^2+^ binding as compared with Aβ_1−x_ and in a manner that does not produce ROS.

## Is there mislocalized copper in the AD brain?

The meta-analysis conducted by Schrag et al. (2011) identified a citation bias in the reporting of metal levels in the brain in AD. Despite the large heterogeneity in the published data, they noted that “this bias was particularly prominent among narrative review articles” and further identified problems with a number of studies. In particular, they noted the report by Markesbery and co-workers (Lovell et al., 1998) was discordant with other studies yet “is the most cited paper on the subject of copper in AD and appears to be the source for numerous articles reporting that copper levels are (several fold) increased in AD” (Schrag et al., 2011). It was also discordant with Markesbery and co-workers' earlier study that reported a significant decrease in copper in AD hippocampus and amygdala (Deibel et al., 1996). Upon exclusion of all studies with methodological shortcomings, the meta-analysis of Schrag et al. (2011) indicated that there was no change in neocortical iron and a significant decrease in neocortical copper in AD as compared with age-matched control tissue.

Since the influential publication of Lovell et al. (1998), contrasting conclusions have been drawn regarding the relationship between copper and AD. Singh et al. (2013) demonstrated a relationship between increased copper levels in brain capillaries and reduced Aβ clearance across the blood–brain barrier (BBB) in normal mice. An X-ray fluorescence (XRF) microscopy study of tissue from two neuropathologically confirmed cases of AD reported “hot-spots” with colocalization of copper and zinc with regions of thioflavin-reactive amyloid (Miller et al., 2006). James et al. (2012) “found no difference in the Cu content of AD samples relative to healthy tissues,” but instead “an increase in the labile pool” of copper within the AD cortex, which they attributed to “a global distortion of brain Cu metabolism in AD, distinct from the formation of insoluble Cu–Aβ” Increased labile copper outside the CNS has also been reported (Squitti et al., 2014a) with increased concentrations of labile (non-ceruoplasmin) Cu^2+^ in serum as a predictor of transitioning from mild cognitive impairment to AD (Squitti, 2014). Rembach et al. (2013) concluded that the previously reported decrease in neocortical copper in AD (Schrag et al., 2011) could be attributed to a reduction in content harbored within soluble extractable tissue from AD frontal cortex.

Animal models have not provided any clear evidence for copper imbalance. The copper in plaques of AD transgenic mice, quantified by XRF microscopy, appears consistent with that of the surrounding neuropil after accounting for local tissue density (James et al., 2017). Leskovjan et al. (2009) argued that failure to observe increased levels of Cu in plaques within APP transgenic mouse models of AD is due to inadequate time for plaques to “sink” this metal within their shorter lifespan and that this is consistent with the absence of neurodegeneration in those models (Bourassa and Miller, 2012). While there appears to be a relationship between copper and APP, the variability between transgenic animal models expressing human APP likely makes them unsuitable for elucidating the association (White et al., 1999; Maynard et al., 2002; Bayer and Multhaup, 2005; Wang et al., 2012; Singh et al., 2013) and similar limitations may also apply to copper and Aβ.

While Szabo et al. (2015) measured no difference in copper levels in the frontal cortex of control and AD subjects, the authors did not rule out the possibility of differences in its cellular localization and chemical speciation. In a similar vein, Bush and coworkers have asserted that “metals both accumulate in microscopic proteinopathies, and can be deficient in cells or cellular compartments. Therefore, bulk measurement of metal content in brain tissue samples reveal only the “tip of the iceberg,” with most of the important changes occurring on a microscopic and biochemical level” (Barnham and Bush, 2014). They further argue that “Zn and Cu are sequestered into plaques, whereas intraneurally these metals are depleted” (Ayton et al., 2013).

The evidence to support an intracellular depletion of copper and zinc in AD remains unclear, although the genesis for this idea appears to have emanated from (i) studies demonstrating that APP overexpression causes copper efflux and intracellular depletion (Treiber et al., 2004; Bayer and Multhaup, 2005), and (ii) the large reported ionophore action the 8-hydroxyquinoline (8HQ)-based compounds 5-chloro-7-iodo-8-hydroxyquinoline (CQ; Treiber et al., 2004; White et al., 2006; Crouch et al., 2009), and 5,7-dichloro-2-[(dimethylamino)methyl]-8-hydroxyquinoline (PBT2), (Adlard et al., 2008, 2011; Crouch et al., 2009, 2011), resulting in dramatic increases in intracellular copper and zinc. In yeast models, an approximate 100-fold increase in intracellular copper levels was reported in response to combined CQ/Cu^2+^ treatment, as compared with a ~10-fold increase following exogenous addition of Cu^2+^ alone (Treiber et al., 2004). These observations were recapitulated in other cell models (CHO and N2a cells expressing APP), again with a 100-fold increase in intracellular copper concentration as compared with basal levels when 10 μM Cu^2+^/CQ was added to culture media, and 10-fold increases in zinc and iron in response to exogenous application of their respective CQ complexes (White et al., 2006).

Given the magnitude of the reported ionophore effect, one might expect a 100-fold increase in intracellular copper levels to place significant stress on cultured cells, especially since other significant cellular events such as neuronal differentiation result in only a 2- to 3-fold increase in copper (no change in iron, manganese, zinc; Ogra et al., 2016). In studies of CQ, the authors concluded there was “no evidence of increased cell death after 6 h of exposure to CQ and metals” (White et al., 2006), although no dose response curve monitoring functional changes (e.g., cell metabolism, caspase activation, direct measures of ROS production) was presented in order to substantiate this. Subsequent studies using the SH-SY5Y neuroblastoma cell line again demonstrated dramatic metal influx in response to treatment with CQ (Crouch et al., 2009, 2011) and also PBT2 (Crouch et al., 2011), an effect that also resulted in greater phosphorylated GSK-3β (pGSK3β). No dose response curves were provided in either of these studies to determine whether the applied concentrations of the 8HQs were toxic. Moreover, there is no reason to assume that basal intracellular metal levels in these cell lines represent a phenotype of copper depletion, since metal levels were reported only as a percentage increase relative to untreated cells (White et al., 2006; Crouch et al., 2009, 2011). An increase in pGSK3β following treatment with this compound may therefore be part of an apoptotic signaling cascade rather than promoting cell survival (Jacobs et al., 2012). Indeed, studies using a homologous terdentate 8HQ resulted in a significant increase in pGSK 3β only at cytotoxic concentrations (Haigh et al., 2016). In this regard, it is noteworthy that Adlard et al. (2011) did perform a dose response and used 100-fold lower PBT2 concentrations, in which case no signaling cascade involving GSK3 phosphorylation was reported.

In summary, there remains a pervasive belief that copper levels are many-fold higher in AD. Some authors have replaced this picture with one incorporating an increase of extracellular/intracellular copper ratio, although this appears to be motivated by reports of ionophore action of certain chelators, coupled with an underlying presumption that they are effective treatments for AD.

## Is therapeutic chelation efficacious?

A large number of metal chelators have been proposed as therapeutics for AD (e.g., Telpoukhovskaia and Orvig, 2013; Robert et al., 2015), while only a handful have been clinically trialed. The most widely promoted therapeutic chelators for AD therapy are CQ (PBT1) and PBT2, both of which are based upon old chemistry with diverse applications (Gholz and Arons, 1964; Stevenson and Freiser, 1967; Rajagopalan et al., 2001; Ding et al., 2005). Terdendate ligands (L) such as PBT2 can bind in a 1:1 (CuL) and a distorted 5-coordinate 1:2 (CuL_2_) form (Kenche et al., 2013), although the predominant Cu^2+^-bound form of this class of terdentate 8HQ in a biological context is predicted to be a ternary (mixed-ligand) metal complex involving His side chains of proteins and peptides (Kenche et al., 2013). While CuL and CuL_2_ are not capable of generating hydroxyl radicals in the presence of the biological reductant such as ascorbate, the dominant ternary metal complexes can produce as many hydroxyl radicals as Cu(Aβ_1−x_) *in vitro* (Mital et al., 2016) and ROS production can be observed following addition of such 8HQs to neural stem cell cultures (Haigh et al., 2016). These observations contrast with the founding principle of therapeutic chelation (Barnham and Bush, 2014).

To distinguish bulk chelation therapy, generally associated with systemic removal of heavy metal toxins from the body, therapeutic chelators have been re-branded as “metal-protein attenuating compounds (MPACs),” “ionophores,” and “metallochaperones.” The use of “MPAC” was popular due to the belief that CQ and PBT2 disaggregated Aβ plaques loaded with Cu^2+^ and Zn^2+^ (Cherny et al., 1999; Lannfelt et al., 2008). When large cellular metal uptake was reported *in vitro*, the term “ionophore” was applied (Treiber et al., 2004; White et al., 2006; Adlard et al., 2008, 2011), while the term “metallochaperone” now tends to be used most often even though the fate of the ligand remains unknown. It is possible 8HQs behave as carrier ionophores within the hydrophobic lipid environment of various cellular membranes, that copper is not released at all from 8HQ ligands once localized to a lipid bilayer, and that 8HQs interfere with native metal binding sites of key regulatory enzymes (Martirosyan et al., 2006; King et al., 2011; Kawamura et al., 2014) due to ternary complex formation.

Studies in mouse models of AD claimed some promise of therapeutic benefit using 8HQ therapeutic chelators (Cherny et al., 2001; Adlard et al., 2008, 2011). Similar to more conventional therapies targeting Aβ, however, the results of therapeutic chelation have been equally disappointing when translated to human clinical trials. As noted by Relkin following the publication of the results from the first phase IIa trial of PBT2 (Relkin, 2008): “The success or failure of PBT2 is predicated on the validity of two controversial hypotheses of AD pathogenesis. The first is the amyloid hypothesis…[and the] second, and arguably more controversial hypothesis, relates to the role of metal ions in AD. Because many factors affect the accumulation of Aβ, whether the attenuation of the interactions of metal ions with Aβ will be sufficient to alter the course of AD is uncertain.” Previous trials of therapeutic chelation using D-penicillamine provided no evidence of altering clinical progression and was terminated early due to adverse events, leading some to question the scientific rationale for pursuing therapeutic chelation with 8HQs (Squitti et al., 2002). Indeed, independent assessments of the human clinical trials using 8HQs repeatedly concluded between 2006 through to 2014 that there “is no evidence that MPACs (PBT1 or PBT2) are of benefit in Alzheimer's dementia” (Jenagaratnam and McShane, 2006; Sampson et al., 2008, 2012, 2014).

Despite the above cautions, the conjecture that 8HQs were effective in treating AD has been far more prevalent. For example, a *post-hoc* analysis of the 2008 phase IIa trial stated in its title that “PBT2 Rapidly Improves Cognition in Alzheimer's Disease (Faux et al., 2010),” although it appears this claim pertains to earlier transgenic animal studies rather than the clinical trial in question. In 2013, it was claimed that “clinical trials targeting metal interactions with Aβ have all shown benefit for patients” (Ayton et al., 2013), and even after the release of findings from a repeat phase II trial in April 2014,^2^ some researchers were slow to abandon the mantra that CQ and PBT2 have had “positive clinical outcomes” (Barnham and Bush, 2014) and “significant positive effects on cognition” (Ryan et al., 2015). This most recent trial did not meet its primary endpoint (a reduction of amyloid burden as compared with placebo), echoing previous warnings about “plaques not being the optimal marker of therapeutic success” (Gouras and Beal, 2001). Notwithstanding, all secondary endpoints other than safety and tolerability were also missed (no change in cognition, neuronal function, brain volume or patient function). Tetradentate 8HQs with very high Cu^2+^ affinity have been proposed as alternatives to bi- and terdendtate 8HQs. In non-transgenic mice subjected to intracranial injection of human Aβ, both CQ and a tetradentate 8HQ (apparent *K*_d_ = 1.26 × 10^−18^ M for Cu^2+^ at pH 7.4) were shown to reverse a loss of contextual fear conditioning which was reasoned to result from the probable extraction of Cu^2+^ from the injected Aβ and its “return to the normal circulation of copper ions” (Ceccom et al., 2012). Tetradentate 8HQs have not progressed to clinical trials.

In summary, there has been a bias toward reporting outcomes of clinical trials of therapeutic copper chelators as positive and beneficial for patients, which drives the continued screening of new chelators in spite of well-defined targets for metal acquisition and release in AD.

## Concluding remarks

Therapeutic chelation in its original formulation aimed to deliver a ligand to the CNS in order to prevent copper-induced misfolding and ROS production by Aβ_1−x_, thereby reducing amyloid deposition and oxidative stress within the AD brain. The concept that metal ion binding to Aβ is responsible for potentiating its toxicity has led to hundreds of *in vitro* studies devoted to investigating the nature of this binding interaction, the mechanism of ROS production and the effects on Aβ aggregation. These studies continue unabated, despite convincing *in vivo* evidence for a direct copper-Aβ interaction or other specific targets for therapeutic chelators, which have so far failed to modify disease outcomes.

The variability in experimental data and their interpretation pertaining to copper speciation and localization has transformed what began as a well-defined objective of inhibiting metal-Aβ interactions into an ill-defined target for therapeutic intervention. A global distortion of copper metabolism in the form of a reduction in copper binding affinity (greater lability) will affect all copper-binding proteins. Hence, it is not clear how the general movement of metal ions, for example from extracellular to intracellular location, will address the underlying cause of the proposed imbalance. Recent first principles calculations taking into account the stochastic nature of copper-Aβ interactions, transient metal release and reuptake, and the finite volume of a typical synapse, also predict that if soluble Aβ oligomers are indeed toxic and Cu^2+^-inducible, then “a partial Cu(II) depletion [by therapeutic chelation] might actually accelerate rather than eliminate the neurotoxic Aβ dimer formation” (Goch and Bal, 2017).

If reports about enrichment of amyloid plaques with Cu^2+^ can be substantiated, then the very high Cu^2+^ affinity of the abundant Aβ_4−x_ isoform may provide a logical interpretation for such enrichment, since it has 3,000-fold higher affinity than Aβ_1−x_ isoforms. One could argue hippocampal Aβ_4−42_ contributes to AD due to the possibility this isoform can accumulate Cu^2+^, although Cu(Aβ_4−42_) does not appear capable of generating ROS. It is now established that endopeptidases such as NEP and IDE can hydrolyse the Glu3-Phe4 bond to generate Aβ_4−x_ and there is a clear inverse correlation between *in vivo* NEP activity/levels and AD. In general agreement with an amyloid cascade hypothesis, a decline or impairment of Aβ-clearance mechanisms in age or AD may result in accumulation of Aβ_1−42_, leaving only the far N-terminus accessible for cleavage yielding detectable post-mortem levels of Aβ_4−42_ and increased levels of this peptide in AD hippocampus. Alternatively, if Aβ_4−42_ or shorter, soluble and transient Aβ_4−x_ proteolytic fragments have a functional role in copper homeostasis, any Cu^2+^ imbalance that might exist in AD or during aging could be associated with a downstream loss of function rather than a gain of toxic function. From this perspective, immunization strategies targeting both Aβ_1–*x*_, and especially its N-truncated isoform (Bayer and Wirths, 2014; Antonios et al., 2015), may perturb such putative function. Considering the relative affinities of Aβ_4−x_ vs. Aβ_1−x_ and that APP was proposed to be a Cu chaperone/transporter despite a modest affinity (apparent *K*_d_ ~ 10 nM at pH 7) copper binding domain (Barnham et al., 2003b; Treiber et al., 2004; Kong et al., 2008), a comparable role for Aβ_4−x_ is not unreasonable. While *in vivo* relevance remains speculative, the irony with respect to the metals hypothesis is that the brain might administer its own therapeutic chelator in the course of the normal catabolism of Aβ_1−x_, and thus restoration of endoproteolytic processing could also restore copper homeostasis.

## Author contributions

The author confirms being the sole contributor of this work and approved it for publication.

### Conflict of interest statement

The author declares that the research was conducted in the absence of any commercial or financial relationships that could be construed as a potential conflict of interest.

## Footnotes

^1^Whilst making for a memorable title, “The galvanization of β-amyloid…”, implies a non-physiological two-electron reduction of Zn^2+^(aq) to Zn(s) (*E*^0^ = −0.76 V vs. SHE).

^2^www.alzforum.org/news/research-news/pbt2-takes-dive-phase-2-alzheimers-trial (1 April 2014).
